# Supplementary material for: The impact of adjuvant chemotherapy on survival in mucinous and non-mucinous rectal adenocarcinoma patients after TME surgery
Source: PLoS One. 2023 Feb 27;18(2):e0282211. doi: 10.1371/journal.pone.0282211 (PMC9970087; doi:10.1371/journal.pone.0282211)
Supplement: S1 File — Data used in the study analyses. (PDF) [file pone.0282211.s001.pdf]

	Sex	Age at diagnosis	pT stage	pN stage	TNM 6	Distance to anal verge cm	Differentiation grade	Vascular invasion	perineuvral growth	MAC yes or no	Overall survival months diagnosis date	Overall survival censored
1	1	89	T3	N2	4	4	2	0	0	0	4	1
2	2	88	T3	N0	1	7	2	0	0	1	13	1
3	2	88	T3	N0	1	6	2	0	0	0	38	1
4	1	90	T3	NX	1	4	2	0	0	0	21	1
5	1	85	T3	N2	4	2	2	0	0	0	9	1
6	1	88	T3	N0	1	14	2	0	0	0	118	1
7	2	84	T3	N0	1	5	2	0	0	0	48	1
8	1	86	T4	N0	1	7	2	0	0	0	17	1
9	2	83	T4	N0	5		1	0	1	0	20	1
10	2	88	T3	N0	2	4	2	1	1	0	19	1
11	2	95	T3	N0	1	1	3	1	0	1	49	1
12	2	88	T3	N1	3	11	2	1	0	0	12	1
13	1	86	T3	N0	1	9	2	0	0	0	171	0
14	2	86	T3	N2	4	13	3	1	1	1	33	1
15	2	82	T3	N0	1	14	2	0	0	0	74	1
16	1	87	T3	N0	1	5	2	0	0	0	107	1
17	2	86	T3	N2	4	4	2	0	0	0	32	1
18	2	87	T3	N0	1	4	2	0	0	0	83	1
19	2	81	T3	N2	4	5	3	0	0	0	27	1
20	2	87	T3	N0	1	12	2	0	0	0	81	1
21	1	82	T3	N2	4	7	3	0	0	0	17	1
22	1	86	T3	N2	4	5	2	0	1	0	77	1
23	2	79	T3	N0	1	4	2	0	0	0	47	1
24	2	82	T3	N0	1	4	3	0	0	0	106	1
25	1	87	T3	N2	4	15	2	1	1	0	57	1
26	1	80	T3	N0	1	20	2	0	0	1	17	1
27	1	80	T3	N0	1	20	2	0	0	0	26	1

28	2	80	T3	N0	1	2	2	0	0	0	75	1
29	2	82	T3	N2	5	7	3	1	0	0	6	1
30	1	84	T4	N1	3	3	2	1	1	0	29	1
31	2	83	T2	N2	5	5	3	0	0	1	32	1
32	1	83	T3	N0	1	10	2	0	0	0	49	1
33	2	83	T2	N1	2	12	2	0	0	0	4	1
34	1	79	T3	N0	1	0	3	0	0	0	117	1
35	1	83	T3	N2	4	5	2	0	0	0	12	1
36	1	78	T3	N2	4	9	3	0	0	0	30	1
37	1	85	T3	N0	1	10	2	0	0	0	24	1
38	1	86	T3	N1	3	12	3	0	0	1	10	1
39	2	77	T3	N0	1	15	2	0	0	0	207	0
40	2	77	T3	N0	5		2	1	0	1	1	1
41	1	78	T3	N2	5	7	3	1	1	0	5	1
42	1	79	T3	N2	5	10	3	1	1	0	5	1
43	2	86	T3	N0	1	14	3	1	1	0	38	1
44	2	80	T3	N2	4	6	3	0	0	1	8	1
45	2	84	T3	N0	1	13	1	0	0	0	35	1
46	2	90	T3	N2	5	4	2	0	0	1	3	1
47	1	77	T3	N2	5	5	2	0	0	0	11	1
48	1	84	T4	N0	1	0	3	1	1	0	37	1
49	1	79	T3	N1	3	5	3	0	1	0	109	1
50	2	77	T3	N1	3	2	3	0	0	1	14	1
51	1	78	T3	N0	1		2	0	0	0	77	1
52	2	81	T3	N0	1	11	2	0	0	0	134	1
53	2	77	T3	N1	3	8	2	0	0	0	56	1
54	1	78	T3	N0	1	0	2	0	0	0	25	1
55	1	77		NX	5	13	3	0	0	0	2	1
56	1	81	T2	N0	5	6	3	0	0	0	5	1
57	2	75	T3	N0	1	8	2	0	0	0	50	1
58	1	75	T2	N1	2	13	2	0	0	0	130	1
59	1	82	T3	N2	4	7	2	0	0	0	32	1
60	1	76	T4	N0	1	2	2	0	0	0	186	0
61	1	82	T3	N1	3	7	2	0	0	0	59	1

62	1	78	T3	N0	1	5	2	0	0	0	140	1
63	2	84	T3	N0	1	10	1	0	0	0	205	0
64	2	78	T3	N0	1	12	2	0	0	0	30	1
65	2	79	T3	N0	1	10	3	0	0	0	69	1
66	1	82	T3	N1	3	14	2	0	1	0	102	0
67	2	78	T3	N0	5	5	2	0	0	0	14	1
68	1	80	T3	N0	1	11	2	0	0	0	56	1
69	2	80	T3	N1	3	0	3	0	0	1	29	1
70	2	75	T3	N2	4	25	2	0	1	0	191	0
71	2	77	T3	N2	5	7	2	0	0	0	63	1
72	2	79	T3	N2	4	6	2	1	1	0	19	1
73	2	76	T4	N0	1	6	2	0	0	0	35	1
74	1	78	T3	N0	1	10	2	0	0	0	114	1
75	1	79	T3	N0	1	12	2	0	0	0	135	1
76	1	79	T3	N0	1	10	2	0	0	0	12	1
77	2	77	T3	N0	1	14	2	0	0	0	166	1
78	1	76	T3	N0	5	12	3	0	0	0	5	1
79	2	81	T3	N0	1	4	3	1	1	0	113	0
80	2	81	T3	N1	3	5	2	1	0	0	75	0
81	1	78	T3	N0	1	13	2	0	0	0	157	0
82	1	76	T3	N0	1	2	3	0	0	0	12	1
83	2	81	T4	N2	4	15	3	1	1	1	22	1
84	1	76	T3	N0	1	10	2	0	0	0	152	0
85	1	72	T2	N2	3	4	3	0	0	0	190	0
86	1	73	T4	N2	5	6	3	1	0	0	7	1
87	1	71	T4	N2	4	2	2	1	0	0	208	0
88	2	78	T2	N1	2	9	3	0	0	0	36	1
89	1	73	T3	N2	4	11	3	0	0	0	188	0
90	2	75	T3	N0	1	11	3	0	0	1	172	0
91	2	76	T4	N0	1	1	2	0	0	0	2	1
92	1	79	T2	N0	4	1	3	0	0	1	112	0
93	2	71	T3	N0	1	12	2	0	0	0	134	1
94	2	80	T4	N0	5	7	2	1	1	0	24	1
95	2	74	T3	N2	5	3	2	1	0	0	38	1

96	1	80	T2	N1	4	10	2	1	0	0	91	0
97	1	78	T3	N0	1	10	2	0	0	0	126	0
98	2	78	T4	N2	4	0	3	1	1	0	12	1
99	2	70	T4	N0	5	2	2	1	1	0	39	1
100	2	71	T3	N2	4	12	2	1	0	0	1	1
101	2	77	T3	N0	1	13	2	0	0	0	75	1
102	1	78	T3	N0	1	10	3	0	0	1	51	1
103	2	75	T3	N0	1	12	2	0	0	0	44	1
104	1	78	T4	N2	4	10	3	0	0	1	64	1
105	1	79	T2	N1	4	4	2	1	0	0	46	1
106	2	76	T3	N2	4	4	2	0	0	0	23	1
107	2	78	T3	N2	4	13	2	1	1	0	72	1
108	2	76	T3	N0	1	2	3	1	1	0	138	0
109	2	74	T3	N1	3	5	2	0	0	0	32	1
110	1	74	T4	N1	5	10	2	0	0	0	3	1
111	1	71	T3	N0	1	2	2	0	0	0	177	1
112	2	71	T3	N1	3	7	2	0	0	0	147	1
113	2	71	T3	N0	1	15	2	0	0	0	72	1
114	1	73	T4	N1	5	2	2	0	0	0	36	1
115	1	75	T4	N1	5	10	2	0	0	1	37	1
116	2	75	T3	N0	1	7	3	0	0	1	141	0
117	2	74	T3	N1	4	6	2	1	1	0	51	1
118	2	77	T3	N2	4	10	3	1	1	0	109	0
119	2	75	T3	N0	1	9	3	0	0	0	138	0
120	2	77	T3	N0	1	8	2	0	0	0	35	1
121	1	77	T3	N0	1	15	2	0	0	0	124	0
122	1	72	T3	N0	1	10	3	0	0	0	162	0
123	2	74	T4	N2	5	18	3	0	0	0	133	1
124	2	72	T3	N0	1		3	0	0	0	110	1
125	1	76	T3	N1	3	12	2	1	0	0	77	1
126	2	72	T1	N0	1	4	2	0	0	0	152	0
127	2	69	T3	N0	1	10	1	0	0	0	194	0
128	2	73	T3	N0	1	12	2	0	0	0	153	0
129	1	69	T3	N1	3	2	2	0	0	0	73	1

130	1	77	T3	N2	4	3	3	1	1	1	24	1
131	1	71	T3	N1	3	3	3	0	0	1	33	1
132	2	71	T3	N2	4	8	2	0	0	0	11	1
133	2	76	T3	N0	1	13	2	0	0	0	116	0
134	1	72	T4	N2	5	13	3	0	0	0	9	1
135	2	77	T3	N1	4	14	3	1	1	0	98	0
136	2	73	T3	N0	1	8	1	0	0	0	188	0
137	2	74	T3	N2	4	4	2	0	0	0	126	0
138	2	73	T2	N2	3	4	2	0	0	0	46	1
139	2	71	T3	N2	4	10	3	1	0	1	16	1
140	2	73	T3	N2	4	9	2	0	0	0	96	1
141	1	72	T3	N0	1	2	3	1	0	0	40	1
142	1	74	T3	N0	1	14	2	0	0	0	117	0
143	2	72	T4	N1	5	2	3	0	1	0	47	1
144	2	68	T3	N0	1	17	2	0	0	0	85	1
145	1	74	T3	N1	3	10	3	1	0	0	129	0
146	2	76	T3	N0	1	10	2	1	0	0	111	0
147	1	69	T3	N1	3	14	3	0	0	1	25	1
148	2	69	T3	N2	4	12	3	0	0	0	157	1
149	1	74	T3	N1	3	12	2	0	0	0	65	1
150	2	73	T3	N0	1	4	2	0	0	0	140	0
151	2	73	T3	N0	1	7	2	0	0	0	123	0
152	2	72	T2	N1	2	13	2	0	0	1	100	1
153	1	75	T3	N0	1	11	2	0	0	0	102	0
154	2	75	T3	N1	3	5	3	0	1	0	110	0
155	2	68	T3	N2	5	8	2	1	0	1	20	1
156	2	67	T3	N2	5	3	3	1	0	0	5	1
157	2	70	T4	N0	1	8	2	0	0	0	19	1
158	2	74	T3	N0	1	7	3	0	0	1	105	0
159	2	67	T3	N0	1	2	2	1	1	0	99	1
160	1	74	T3	N1	3	6	3	1	1	0	51	1
161	2	71	T3	N2	4	10	2	1	1	0	15	1
162	1	71	T2	N1	4		3	1	0	0	60	1
163	2	66	T2	N2	3	6	2	0	0	0	10	1

164	1	73	T4	N1	3	13	2	0	1	0	60	1
165	2	72	T4	N2	4	12	3	0	0	0	29	1
166	2	67	T3	N0	1	5	2	1	0	0	185	0
167	1	71	T3	N0	1	7	2	1	1	0	125	1
168	1	71	T3	N0	1	6	2	0	0	0	42	1
169	2	70	T3	N0	1	10	2	0	0	0	149	0
170	1	68	T3	N2	5	0	3	0	0	1	47	1
171	2	70	T3	N2	4	2	3	0	0	0	73	1
172	2	65	T3	N0	1	3	2	0	0	1	37	1
173	2	76	T3	NX	1	14	2	0	0	0	159	1
174	1	71	T3	N2	4	4	2	0	1	0	39	1
175	2	72	T3	N2	4	1	2	0	0	0	60	1
176	2	68	T3	N0	1	13	2	0	0	0	26	1
177	2	71	T3	N1	3	8	2	1	0	0	127	0
178	2	68	T3	N0	1	5	2	0	0	0	88	1
179	2	74	T2	N1	3	0	3	1	1	0	38	1
180	1	66	T3	N1	3	10	2	0	0	0	2	1
181	2	71	T3	N0	1	13	2	0	0	0	28	1
182	1	70	T3	N1	3	15	2	0	0	0	36	1
183	2	70	T3	N2	5	11	3	1	1	0	3	1
184	1	71	T1	N0	3	10	3	0	1	0	40	0
185	2	66	T3	NX	1	5	3	0	0	1	176	0
186	1	68	T3	N1	3	10	3	0	0	0	28	1
187	2	66	T3	N2	5	6	2	1	1	0	172	0
188	2	70	T3	N2	4	9	2	1	0	0	132	0
189	1	65	T3	N1	3	10	3	0	0	0	11	1
190	2	69	T3	N2	4	7	3	0	0	1	138	0
191	2	65	T3	N0	1	12	3	0	0	0	139	0
192	2	68	T4	N0	5	2	2	0	0	0	29	1
193	2	70	T3	N0	1	0	2	0	0	0	130	0
194	2	50	T3	N0	1	5	2	0	0	0	127	0
195	1	68	T3	N0	1	12	2	0	0	0	153	0
196	1	65	T3	N0	5	12	2	0	0	0	179	0
197	1	71	T3	N0	1	10	2	0	0	0	123	0

198	1	69	T4	N1	3	5	3	1	0	1	129	0
199	1	64		NX	5	5	2	0	0	0	14	1
200	2	67	T3	N1	3	0	3	1	0	1	149	0
201	1	71	T3	N0	1	13	2	0	0	0	69	1
202	2	71	T3	N0	1	12	2	0	0	0	108	0
203	2	69	T3	N0	5	10	2	0	0	0	36	1
204	1	67	T2	N1	2	5	3	0	0	0	151	0
205	2	70	T3	N2	4	15	3	1	1	0	44	1
206	2	68	T2	N1	3	7	2	0	0	0	80	1
207	1	71	T3	N2	4	7	3	0	0	0	19	1
208	2	67	T3	N1	5	3	2	0	0	0	25	1
209	2	65	T3	N0	1	9	2	0	0	0	10	1
210	2	68	T3	N0	1	14	3	0	0	0	135	0
211	2	70	T3	N1	5	7	1	0	0	0	98	1
212	1	68	T3	N0	1	1	3	0	0	0	20	1
213	1	66	T2	N2	3	7	3	1	0	0	139	1
214	2	62	T3	N0	1	8	2	0	0	0	198	0
215	2	66	T3	N1	5	10	2	1	0	0	17	1
216	2	70	T3	N1	3	13	2	1	0	0	88	1
217	1	60	T3	N1	3	4	2	0	0	0	153	0
218	2	67	T3	N0	1	2	2	0	0	0	146	0
219	2	64	T3	N0	5	14	2	0	0	0	148	1
220	1	68	T3	N1	3	5	3	0	0	1	124	0
221	2	69	T3	N0	1	8	1	0	1	0	114	0
222	2	68	T3	N2	4	1	2	1	1	0	74	1
223	2	66	T3	N0	1	7	2	0	0	0	153	0
224	2	63	T3	N0	1	10	1	0	0	0	65	1
225	1	69	T3	N0	1	15	2	0	0	1	120	0
226	1	64	T3	N1	3	13	3	1	0	0	57	1
227	2	67	T3	N2	4	8	3	1	1	0	39	1
228	1	68	T3	N0	1	2	2	0	0	0	33	1
229	1	65	T2	N1	2	0	2	0	0	0	150	0
230	1	60	T2	N1	2	5	2	1	0	0	161	0
231	2	66	T3	N1	3	5	3	0	1	0	87	1

232	2	67	T3	N1	3	13	3	0	0	0	131	0
233	1	66	T2	N2	4	2	2	1	0	0	69	1
234	1	67	T3	N1	5	8	3	0	0	1	28	1
235	2	65	T3	N1	3	1	3	0	0	0	35	1
236	1	63	T3	N2	5	6	3	1	1	0	8	1
237	2	66	T3	N1	3	4	3	0	0	1	19	1
238	1	66	T3	N1	5	8	2	1	0	0	52	1
239	2	66	T2	N1	2	5	2	1	1	0	134	0
240	2	68	T3	N0	1	1	3	0	1	0	124	0
241	2	64	T3	N0	1	13	2	0	0	0	151	0
242	1	65	T3	N2	4	6	3	1	0	1	144	0
243	1	68	T2	N1	2	10	2	0	1	0	111	0
244	2	65	T3	N0	1	13	2	0	1	0	139	0
245	2	65	T3	N0	1	8	2	0	0	0	140	0
246	2	66	T4	N1	4	12	2	1	0	0	125	0
247	1	63	T4	N0	1	7	2	0	0	0	33	1
248	1	66	T2	N1	2	8	2	0	0	0	133	0
249	2	64	T3	N1	5	9	3	0	0	0	14	1
250	1	65	T3	N2	4	6	2	0	0	0	32	1
251	2	65	T3	N2	4	14	3	1	1	0	6	1
252	2	59		NX	5	4	2	0	0	0	24	1
253	1	67	T3	N1	3	9	2	0	1	0	113	0
254	2	64	T2	N1	4	9	2	0	0	0	143	0
255	2	64	T3	N2	4	15	2	0	0	1	30	1
256	1	64	T4	N1	3	0	3	0	0	0	116	1
257	1	68	T2	N2	4	12	2	1	0	1	94	0
258	2	61	T3	N1	3	13	2	0	0	0	94	1
259	2	66	T3	N1	3	11	2	1	0	0	33	1
260	1	61	T3	N0	1	4	2	0	0	0	172	0
261	1	65	T2	N2	3	4	2	0	0	0	131	0
262	2	64	T2	N1	2	13	2	0	0	0	122	0
263	2	67	T3	N0	1	13	2	1	0	0	22	1
264	1	66	T4	N1	3	8	3	1	1	0	122	0
265	2	66	T3	N0	5	12	3	1	1	1	34	1

266	1	63	T3	N0	1	13	2	0	1	0	151	0
267	1	66	T3	N0	1	11	2	0	0	0	121	0
268	2	65	T3	N0	1	9	2	0	0	0	122	0
269	2	66	T4	N2	4	5	3	1	1	0	8	1
270	1	62	T3	N0	1	7	2	0	0	1	99	1
271	1	61	T3	N1	3	4	2	0	0	0	95	1
272	2	66	T3	N0	1	13	3	1	0	0	106	0
273	2	62	T3	N1	5	12	2	0	0	0	31	1
274	2	62	T3	N0	5	12	2	1	1	1	142	0
275	1	65	T3	N2	4	11	3	1	1	0	105	0
276	2	65	T3	N0	1	11	2	0	0	0	109	0
277	2	63	T4	N0	1	3	3	0	0	0	34	1
278	1	57	T3	N1	3	11	3	1	0	0	26	1
279	2	61	T3	N0	1	5	3	0	0	1	155	0
280	2	65	T3	N0	1	13	3	0	1	0	111	0
281	1	61	T4	N2	5	13	3	0	0	0	150	0
282	2	59	T3	N1	3	7	2	0	0	0	131	1
283	2	62	T3	N2	5	9	2	1	1	0	47	1
284	1	65	T3	N0	5	4	3	1	0	0	37	1
285	1	64	T3	N1	3	1	2	0	0	0	27	1
286	2	63	T3	N1	3	10	2	1	0	0	30	1
287	1	59	T3	N0	1	9	1	0	0	0	168	0
288	2	58	T3	N0	1	3	2	0	0	0	179	0
289	1	60	T3	N2	5	9	2	1	0	0	10	1
290	2	56	T3	N0	5	5	2	0	0	0	30	1
291	1	64	T2	N1	2	11	2	1	0	0	99	0
292	2	59	T3	N2	4	7	2	1	0	0	159	0
293	2	64	T3	N1	2	10	3	1	1	0	98	1
294	1	56	T4	N1	4	4	3	1	0	0	32	1
295	2	61	T3	N0	1	9	3	0	0	0	138	0
296	1	58	T2	N2	3	13	3	1	0	0	163	0
297	1	60	T4	N0	1	1	3	0	0	1	147	0
298	2	59	T3	N2	2	15	2	0	0	0	105	1
299	2	56	T3	N0	1	12	2	0	0	0	187	0

300	2	56	T2	N2	5	5	2	0	0	0	19	1
301	1	62	T3	N2	4	14	2	0	0	0	112	0
302	2	63	T3	N0	1	15	2	0	0	0	107	0
303	2	58	T4	N2	5	5	2	1	1	0	18	1
304	2	61	T4	N3	4	12	2	0	0	0	116	0
305	2	57	T3	N1	3	12	3	1	0	0	26	1
306	1	55	T3	N0	1	6	2	0	0	1	43	1
307	1	60	T3	N2	4	5	3	1	0	0	47	1
308	2	56	T3	N2	4	5	3	1	0	1	163	0
309	2	55	T3	N1	3	4	3	0	0	0	190	0
310	1	62	T3	N2	5	2	2	0	0	0	43	1
311	2	59	T3	N0	1	13	2	0	0	0	127	0
312	2	60	T4	N0	4	10	2	0	0	0	122	0
313	2	61	T3	N0	1	10	2	0	0	0	103	0
314	2	60	T3	N0	1	13	2	0	0	0	107	0
315	2	55	T3	N2	4	2	2	0	0	0	170	0
316	1	61	T3	N0	1	6	2	0	0	0	103	0
317	1	60	T3	N0	1	1	3	1	1	1	30	0
318	2	59	T3	N0	1	15	2	0	1	0	106	0
319	1	58	T3	N2	4	7	3	1	0	0	82	1
320	1	56	T3	N0	1	13	2	0	0	0	133	0
321	1	57	T3	N0	1	5	3	0	1	0	61	1
322	2	56	T3	N0	1	4	2	0	0	0	76	1
323	2	52	T3	N0	1	10	2	0	0	0	167	0
324	1	55	T3	N0	1	13	2	0	0	0	135	0
325	1	57	T3	N0	4	10	2	1	1	0	104	0
326	1	54	T3	N0	1	4	3	1	0	0	146	0
327	2	53	T3	N0	5	11	2	0	0	0	149	0
328	2	53	T1	N1	3	10	2	0	0	0	140	0
329	2	57	T3	N1	2	11	2	0	0	1	98	0
330	1	53	T3	N2	4	3	2	0	1	0	143	0
331	1	50	T3	N0	1	8	2	0	0	0	175	0
332	1	55	T3	N1	3	10	3	1	1	0	90	1
333	1	52	T3	N1	3	13	2	0	0	1	150	0

334	2	52	T3	N2	4	10	2	0	1	0	145	0
335	1	54	T4	N1	4	5	2	1	1	0	15	1
336	1	53	T3	N1	3	13	3	1	0	0	49	1
337	1	54	T3	N0	5	8	2	1	1	0	107	0
338	1	52	T3	N2	4	9	3	0	0	1	138	0
339	1	47	T3	N1	5	9	2	1	0	0	33	1
340	1	46	T3	N1	3	15	3	0	0	1	60	1
341	2	47	T4	N2	5	10	3	0	0	1	8	1
342	1	47	T3	N1	3	10	2	0	0	0	41	1
343	1	52	T3	N2	4	8	3	0	1	0	113	0
344	2	53	T3	N0	1	10	3	1	1	0	100	0
345	1	52	T3	N2	5	10	2	1	0	0	55	1
346	2	49	X	X	5	6	2	0	0	0	133	0
347	2	47	T4	N0	5	8	2	0	0	1	57	1
348	2	50	T3	N1	5	7	2	0	0	0	31	1
349	2	48	T3	N2	4	8	3	0	0	1	33	1
350	2	44	T3	N0	1	4	2	0	1	0	24	1
351	2	47	T3	N2	4	9	2	0	0	0	138	0
352	2	48	T3	N0	1	8	2	0	0	0	122	0
353	1	49	T3	N0	1	14	2	0	0	0	105	0
354	2	48	T3	N1	3	0	3	1	1	0	119	0
355	2	47	T4	N1	3	5	3	1	1	1	12	1
356	2	47	T3	N0	1	0	3	0	0	1	104	0
357	1	45	T4	N2	4	5	3	1	1	0	20	1
358	1	41	T1	N1	2	10	1	0	0	0	147	0
359	1	34	T4	N0	1	7	2	0	0	0	197	0
360	2	38	T3	N2	5	12	2	1	1	0	31	1
361	1	40	T3	N1	3	8	2	0	0	0	114	0
362	2	32	T2	N1	2	10	3	0	0	0	200	0
363	2	33	T3	N1	3	6	2	1	0	0	179	0
364	1	38	T3	N2	4	10	2	0	0	0	79	1
365	2	25	T4	N2	4	1	3	0	0	1	17	1

cancer specific survival months diagnos date	cancer specific survival censored diagnos date	Disese free survival diagnosis date	Disease free survival censored	Distant recurrence free survival diagnosis date	Distan recurrence free survival censored	Local recurrence free survival diagnosis date	Local recurrence free survival censored	Preoperative chemotherapy yes or no	Type of preoperative Chemo	RT yes/no	Short RT vs Long RT	Radical surgery
4	1	4	0	4	0	4	0	0		0	0	0
13	0	13	0	13	0	13	0	0		0	0	0
38	1	38	0	38	0	38	0	0		0	0	0
21	0	21	0	21	0	21	0	0		0	0	1
9	1	7	1	9	0	7	1	0		0	0	0
118	0	118	0	118	0	118	0	0		0	0	0
48	1	48	0	48	0	48	0	0		1	2	0
17	1	12	1	12	1	12	1	0		0	0	1
20	1	0	1			20	0	0		0	0	1
19	0	14	1	14	1	19	0	0		0	0	0
49	0	49	0	49	0	49	0	0		0	0	0
12	0	12	0	12	0	12	0	0		0	0	0
171	0	171	0	171	0	171	0	0		0	0	0
33	0	33	0	33	0	33	0	0		1	1	0
74	0	74	0	74	0	74	0	0		0	0	0
107	0	107	0	107	0	107	0	0		0	0	0
32	1	28	1	32	0	28	1	0		1	1	1
83	0	83	0	83	0	83	0	0		0	0	0
27	1	16	1	16	1	27	0	0		0	0	0
81	0	81	0	81	0	81	0	0		0	0	0
17	1	16	1	16	1	17	0	0		1	1	0
77	1	74	1	74	1	74	1	0		0	0	0
47	0	47	0	47	0	47	0	0		0	0	0
106	0	106	0	106	0	106	0	0		1	1	0
57	0	57	0	57	0	57	0	0		0	0	0
17	1	17	0	17	0	17	0	0		0	0	0
26	1	15	1	15	1	26	0	0		0	0	0

75	0	75	0	75	0	75	0	0		1	2	0
6	1	1	1	1	1	6	0	0		0	0	0
29	1	14	0	14	0	29	0	0		1	1	1
32	1	5	1	5	1	32	0	0		1	2	0
49	0	49	0	49	0	49	0	0		0	0	0
4	1	4	0	4	0	4	0	0		0	0	0
117	0	117	0	117	0	117	0	0		1	1	0
12	1	7	1	12	0	7	1	0		1	1	0
30	1	14	1	30	0	14	1	0		1	1	0
24	0	24	0	24	0	24	0	0		1	1	0
10	0	10	0	10	0	10	0	0		0	0	0
207	0	207	0	207	0	207	0	0		0	0	0
1	1	0	1			1	0	0		0	0	0
5	1	0	1			5	0	0		0	0	0
5	1	0	1			5	0	0		0	0	0
38	0	38	0	38	0	38	0	0		0	0	0
8	1	8	0	8	0	8	0	0		1	2	0
35	0	35	0	35	0	35	0	0		0	0	0
3	1	2	1	2	1	3	0	0		1	1	0
11	1	11	0	11	0	11	0	0		1	1	0
37	0	37	0	37	0	37	0	0		1	2	1
109	0	109	0	109	0	109	0	0		1	1	0
14	1	13	1	13	1	14	0	0		1	1	0
77	0	76	1	76	1	77	0	0		0	0	0
134	1	82	0	82	1	82	1	0		1	1	0
56	0	56	0	56	0	56	0	0		1	1	0
25	0	14	1	14	1	14	1	0		1	1	1
2	1	0	1			2	0	0		0	0	0
5	0	5	0	5	0	5	0	0		1	1	0
50	1	27	1	27	1	50	0	0		1	2	0
130	0	87	1	87	1	130	0	0		0	0	0
32	1	5	1	5	1	32	0	0		1	1	0
186	0	186	0	186	0	186	0	0		1	2	0
59	0	59	0	59	0	59	0	0		0	0	0

140	0	140	0	140	0	140	0	0		1	1	0
205	0	205	0	205	0	205	0	0		1	1	0
30	0	30	0	30	0	30	0	0		0	0	0
69	0	69	0	69	0	69	0	0		1	1	0
102	0	102	0	102	0	102	0	0		0	0	0
14	1	0	1			14	0	1	2	0	0	0
56	0	56	0	56	0	56	0	0		1	1	0
29	1	14	1	14	1	29	1	0		0	0	0
191	0	191	0	191	0	191	0	0		0	0	0
63	1	14	1	14	1	63	0	1	0	0	0	0
19	1	17	1	17	1	19	0	0		1	1	0
35	0	2	1	2	1	6	1	1	0	0	0	0
114	0	14	1	14	1	114	0	0		1	1	0
135	0	135	0	135	0	135	0	0		0	0	0
12	0	12	0	12	0	12	0	0		1	1	0
166	0	166	0	166	0	166	0	0		1	1	0
5	1	2	1	2	1	5	0	0		1	1	0
113	0	113	0	113	0	113	0	0		1	1	0
75	0	28	1	28	1	75	0	0		1	1	0
157	0	157	0	157	0	157	0	0		0	0	0
12	0	12	0	12	0	12	0	0		1	1	0
22	1	19	1	19	0	19	1	0		0	0	1
152	0	152	0	152	0	152	0	0		1	1	0
190	0	190	0	190	0	190	0	0		0	0	0
7	1	3	1	3	1	7	0	0		1	2	0
208	0	208	0	208	0	208	0	0		1	1	1
36	1	14	1	14	1	36	0	1	2	0	0	0
188	0	188	0	188	0	188	0	0		0	0	0
172	0	172	0	172	0	172	0	0		1	1	0
2	1	2	0	2	0	2	0	0		0	0	1
112	0	13	1	13	1	112	0	0		0	0	0
134	0	134	0	134	0	134	0	0		1	1	0
24	1	8	1	8	1	24	0	0		0	0	1
38	1	2	1	2	1	24	1	0		0	0	0

91	0	91	0	91	0	91	0	0		0	0	0
126	0	126	0	126	0	126	0	0		1	1	0
12	1	12	1	12	1	12	0	0		1	1	1
39	1	0	1			39	0	0		1	2	0
1	0	1	0	1	0	1	0	0		1	1	0
75	1	50	1	50	1	75	0	0		1	1	0
51	0	51	0	51	0	51	0	0		0	0	0
44	0	44	0	44	0	44	0	0		1	1	0
64	1	6	1	64	1	6	1	0		0	0	1
46	0	46	0	46	0	46	0	0		0	0	0
23	1	17	1	23	0	17	1	1	1	1	2	0
72	0	72	0	72	0	72	0	0		1	1	0
138	0	138	0	138	0	138	0	0		1	1	0
32	0	32	0	32	0	32	0	0		0	0	0
3	1	0	1			3	0	0		1	1	0
177	0	177	0	177	0	177	0	0		1	1	0
147	0	147	1	147	0	147	1	0		1	1	0
72	1	10	1	10	1	72	0	0		0	0	0
36	1	1	1	1	1	36	0	0		1	2	0
37	1	21	1	21	1	37	0	1	0	1	2	1
141	0	141	0	141	0	141	0	1	0	1	2	0
51	1	20	1	20	1	51	0	0		1	2	0
109	0	109	0	109	0	109	0	0		1	1	0
138	0	138	0	138	0	138	0	0		1	1	0
35	0	35	0	35	0	35	0	0		1	1	0
124	0	124	0	124	0	124	0	0		0	0	0
162	0	162	0	162	0	162	0	0		1	2	0
133	0	133	0	133	0	133	0	1	0	0	0	0
110	1	81	1	81	1	110	0	0		1	1	0
77	1	73	1	73	1	77	0	0		1	1	0
152	0	152	0	152	0	152	0	0		1	1	0
194	0	194	0	194	0	194	0	0		1	2	0
153	0	153	0	153	0	153	0	0		1	2	0
73	1	40	1	40	1	73	0	0		1	1	0

24	1	14	1	14	1	24	0	0		1	1	0
33	1	12	1	12	1	33	0	0		1	1	0
11	1	11	0	11	0	11	0	0		1	2	1
116	0	5	1	116	0	5	1	0		1	2	0
9	1	0	1			9	0	0		1	2	1
98	0	39	1	39	1	98	0	0		1	1	0
188	0	188	0	188	0	188	0	0		1	1	0
126	0	126	0	126	0	126	0	0		0	0	0
46	1	39	1	39	1	39	1	0		1	1	0
16	1	11	1	11	1	16	0	0		1	2	0
96	0	96	0	96	0	96	0	0		1	1	0
40	1	34	1	34	1	40	0	0		0	0	0
117	0	117	0	117	0	117	0	0		1	2	0
47	1	4	1	4	1	47	0	1	0	1	2	0
85	0	85	0	85	0	85	0	0		0	0	0
129	0	129	0	129	0	129	0	0		1	1	0
111	0	111	0	111	0	111	0	0		1	1	0
25	1	25	0	25	0	25	0	0		1	2	0
157	0	157	0	157	0	157	0	0		1	1	0
65	0	65	0	65	0	65	0	0		0	0	0
140	0	140	0	140	0	140	0	0		1	2	0
123	0	29	1	29	1	123	0	0		1	2	1
100	0	100	0	100	0	100	0	0		0	0	0
102	0	102	0	102	0	102	0	0		0	0	0
110	0	110	0	110	0	110	0	0	0	1	1	0
20	1	1	1	1	1	20	0	0		1	1	0
5	1	0	1			5	0	0		1	2	0
19	1	10	1	10	1	15	1	0		1	2	0
105	0	105	0	105	0	105	0	0		0	0	0
99	0	90	1	90	1	99	0	0		1	2	0
51	0	51	0	51	0	51	0	0		1	1	0
15	1	13	1	13	1	15	0	0		1	1	0
60	1	59	1	59	1	61	0	0		1	1	0
10	1	10	0	10	0	10	0	0		1	2	0

60	1	31	1	31	1	60	0	0			1	1	1
29	1	25	1	29	0	25	1	0			0	0	1
185	0	185	0	185	0	185	0	0			1	1	0
125	0	125	0	125	0	125	0	0			1	1	0
42	0	42	0	42	0	42	0	0			1	1	0
149	0	149	0	149	0	149	0	0			1	1	0
47	1	0	1			47	0	0			1	1	0
73	1	58	1	58	1	73	0	0			1	1	0
37	0	37	0	37	0	37	0	1	9		1	2	1
159	0	159	0	159	0	159	0	0			1	2	0
39	1	27	1	27	1	39	0	0			1	2	1
60	0	24	1	24	1	60	0	0			1	1	0
26	1	17	1	17	1	26	0	1	0		1	2	1
127	0	127	0	127	0	127	0	0			1	2	0
88	0	88	0	88	0	88	0	0			1	1	0
38	1	8	1	8	1	38	0	0			1	1	0
2	0	2	0	2	0	2	0	0			0	0	0
28	1	28	0	28	0	28	0	1	2		1	2	0
36	0	36	0	36	0	36	0	0			0	0	0
3	1	0	1			3	0	0			1	1	1
40	0	4	1	4	1	40	0	0			0	0	0
176	0	16	1	16	1	102	0	1	0		1	2	0
28	0	26	1	26	1	28	0	0			1	2	0
172	0	11	1	11	1	172	0	1	0		1	2	0
132	0	20	1	20	1	132	0	0			1	1	0
11	1	10	1	11	0	10	1	0			1	1	0
138	0	138	0	138	0	138	0	0			1	1	0
139	0	33	1	139	0	33	1	0			1	2	0
29	1	8	1	8	1	29	0	0			1	2	1
130	0	130	0	130	0	130	0	0			1	1	0
127	0	127	0	127	0	127	0	0			1	1	0
153	0	153	0	153	0	153	0	0			1	1	0
179	0	179	0	179	0	179	0	1	8		1	2	0
123	0	123	0	123	0	123	0	0			1	1	0

129	0	129	0	129	0	129	0	1	0	1	2	0
14	1	0	1			14	0	0		1	2	0
149	0	149	0	149	0	149	0	1	0	1	2	0
69	1	17	1	17	1	69	0	1	1	1	2	0
108	0	108	0	108	0	108	0	0		1	1	0
36	1	8	1	8	1	36	0	1	0	1	1	0
151	0	151	0	151	0	151	0	1	0	1	2	0
44	1	40	0	40	0	44	0	0		0	0	0
80	1	62	1	62	1	62	1	0		1	2	0
19	1	16	1	16	1	19	0	0		1	1	0
25	0	25	1	25	0	22	1	0		1	2	1
10	1	10	0	10	0	10	0	1	0	1	2	0
135	0	135	0	135	0	135	0	0		1	1	0
98	1	17	1	17	1	98	0	1	1	1	1	0
20	1	20	0	20	0	20	0	1	1	1	2	0
139	0	139	0	139	0	139	0	0		1	1	0
198	0	198	0	198	0	198	0	0		1	2	0
17	0	9	1	9	1	17	0	0		1	1	0
88	0	4	1	4	1	88	0	1	0	1	1	0
153	0	153	0	153	0	153	0	0		1	1	0
146	0	146	0	146	0	146	0	0		1	1	0
148	1	64	1	64	1	148	0	0		1	1	0
124	0	4	1	4	1	124	0	1	0	0	0	0
114	0	114	0	114	0	114	0	0		0	0	0
74	0	14	1	14	1	74	0	0		1	1	0
153	0	153	0	153	0	153	0	0		0	0	0
65	0	65	0	65	0	65	0	1	0	1	2	0
120	0	120	0	120	0	120	0	1	1	1	1	0
57	1	55	1	55	1	57	0	0		1	1	0
39	1	13	1	39	0	13	1	0		1	1	0
33	0	33	1	33	1	33	0	1	0	1	1	0
150	0	150	0	150	0	150	0	0		1	1	0
161	0	161	0	161	0	161	0	0		1	1	0
87	1	83	1	83	1	87	0	0		1	1	0

131	0	131	0	131	0	131	0	0		1	1	0
69	0	69	0	69	0	69	0	0		1	1	0
28	1	0	1			28	0	1	1	1	1	0
35	1	25	1	35	0	25	1	1	0	1	2	0
8	1	7	1	7	1	8	0	1	0	1	2	0
19	1	12	1	12	1	14	1	1	0	1	2	1
52	1	30	1	30	1	52	0	0		1	1	0
134	0	134	0	134	0	134	0	0		1	1	0
124	0	124	0	124	0	124	0	0		1	1	0
151	0	151	0	151	0	151	0	0		1	1	0
144	0	144	0	144	0	144	0	1	0	1	2	0
111	0	111	0	111	0	111	0	0		1	1	0
139	0	139	0	139	0	139	0	0		1	1	0
140	0	140	0	140	0	140	0	0		1	2	0
125	0	125	0	125	0	125	0	1	0	1	1	0
33	0	33	0	33	0	33	0	0		1	2	0
133	0	29	1	29	1	133	0	0		1	2	1
14	1	0	1			14	0	0		1	1	0
32	1	17	1	17	1	32	0	1	0	1	2	0
6	1	6	0	6	0	6	0	0		1	1	0
24	1	2	1	2	1	24	0	1	11	1	2	0
113	0	113	0	113	0	113	0	0		1	1	0
143	0	143	0	143	0	143	0	0		1	1	0
30	1	15	1	15	1	30	0	0		1	1	0
116	0	32	1	59	1	32	1	1	0	1	2	0
94	0	94	0	94	0	94	0	0		1	1	0
94	0	94	0	94	0	94	0	0		1	1	0
33	0	33	0	33	0	33	0	0		1	1	0
172	0	9	1	9	1	172	0	0		1	1	0
131	0	131	0	131	0	131	0	0		1	1	0
122	0	122	0	122	0	122	0	0		1	2	0
22	0	15	1	15	1	22	0	0		0	0	0
122	0	122	0	122	0	122	0	1	1	1	2	1
34	1	11	1	11	1	34	0	1	1	1	1	0

151	0	151	0	151	0	151	0	0		1	1	0
121	0	121	0	121	0	121	0	0		0	0	0
122	0	122	0	122	0	122	0	0		1	1	0
8	1	8	0	8	0	8	0	0		1	1	0
99	0	99	0	99	0	99	0	0		1	1	0
95	1	62	1	62	1	95	0	0		1	2	0
106	0	106	0	106	0	106	0	1	1	1	1	0
31	1	4	1	4	1	31	0	1	1	0	0	0
142	0	142	0	142	0	142	0	1	0	1	2	0
105	0	105	0	105	0	105	0	0		1	1	0
109	0	109	0	109	0	109	0	0		1	1	0
34	1	2	1	2	1	34	0	1	1	0	0	1
26	0	26	0	26	0	26	0	0		1	2	0
155	0	7	1	7	1	155	0	0		0	0	0
111	0	111	0	111	0	111	0	0		0	0	0
150	0	150	1	150	1	150	0	1	0	0	0	0
131	0	66	1	66	1	131	0	1	0	1	2	0
47	1	19	1	19	1	47	0	1	1	1	1	0
37	1	0	1			37	0	0		0	0	1
27	1	27	0	27	0	27	0	0		1	2	0
30	1	24	1	24	1	30	0	0		1	1	0
168	0	168	0	168	0	168	0	0		1	1	0
179	0	179	0	179	0	179	0	0		1	1	0
10	1	0	1			10	0	0		0	0	0
30	1	1	1	1	1	30	0	1	10	1	2	1
99	0	99	0	99	0	99	0	0		0	0	0
159	0	159	0	159	0	159	0	0		1	1	0
98	0	32	1	32	1	98	0	0		1	1	0
32	1	11	1	11	1	32	0	0		1	1	1
138	0	138	0	138	0	138	0	0		0	0	0
163	0	163	0	163	0	163	0	0		0	0	0
147	0	147	0	147	0	147	0	0		1	1	0
105	0	105	0	105	0	105	0	0		0	0	0
187	0	187	0	187	0	187	0	0		1	1	0

19	1	0	1			19	0	1	0	1	2	0
112	0	100	0	112	0	112	0	0		1	2	0
107	0	32	0	107	0	107	0	0		1	1	0
18	1	2	1	2	1	12	1	1	1	1	2	1
116	0	116	0	116	0	116	0	0		1	2	0
26	1	6	1	6	1	26	0	1	0	1	1	0
43	1	42	1	43	0	42	1	0		1	2	1
47	1	23	1	23	1	47	0	0		1	1	0
163	0	163	0	163	0	163	0	1	0	1	2	0
190	0	190	0	190	0	190	0	0		1	2	0
43	1	43	1	43	1	43	0	1	13	1	1	1
127	0	127	0	127	0	127	0	0		1	1	0
122	0	122	0	122	0	122	0	1	1	1	2	0
103	0	15	0	103	0	15	1	1	0	1	1	0
107	0	21	1	21	1	107	0	0		0	0	0
170	0	170	0	170	0	170	0	0		1	1	0
103	0	103	0	103	0	103	0	0		1	1	0
30	0	30	0	30	0	30	0	0		1	1	1
106	0	106	0	106	0	106	0	0		1	1	0
82	1	55	1	55	1	55	1	1	0	1	2	0
133	0	133	0	133	0	133	0	1	0	1	2	0
61	1	27	1	27	1	61	0	0		1	1	0
76	1	13	1	13	1	76	0	0		1	1	0
167	0	167	0	167	0	167	0	0		1	1	0
135	0	135	0	135	0	135	0	0		1	1	0
104	0	104	0	104	0	104	0	0		1	1	0
146	0	15	1	146	0	15	1	0		1	1	1
149	0	149	0	149	0	149	0	1	0	1	1	0
140	0	140	0	140	0	140	0	0		0	0	0
98	0	98	0	98	0	98	0	0		1	1	0
143	0	143	0	143	0	143	0	0		1	1	0
175	0	13	1	13	1	175	0	0		1	1	0
90	1	39	1	61	1	39	1	0		1	1	0
150	0	150	0	150	0	150	0	0		1	1	0

145	0	145	0	145	0	145	0	0		1	1	0
15	1	5	1	5	1	15	0	1	0	1	2	0
49	0	49	0	49	0	49	0	0		0	0	0
107	0	107	0	107	0	107	0	1	0	1	1	0
138	0	138	0	138	0	138	0	1	0	1	2	0
33	1	0	1			33	0	0		1	2	0
60	1	12	1	12	1	60	0	0		1	2	0
8	1	4	1	4	1	8	0	0		1	1	1
41	1	5	1	5	1	5	1	0		1	2	0
113	0	113	0	113	0	113	0	0		1	1	0
100	0	14	1	14	1	100	0	0		1	1	0
55	1	14	1	14	1	55	0	1	5	1	1	0
133	0	133	0	133	0	133	0	1	0	1	1	0
57	1	28	1	28	1	57	0	0		1	1	0
31	1	0	1			11	1	0		1	2	0
33	1	25	1	29	1	25	1	1	2	1	1	0
24	1	15	1	24	0	15	1	0		1	1	0
138	0	138	0	138	0	138	0	1	1	1	2	0
122	0	122	0	122	0	122	0	1	1	1	2	0
105	0	105	0	105	0	105	0	0		1	1	0
119	0	119	0	119	0	119	0	1	0	1	2	0
12	1	12	0	12	0	12	0	0		1	2	1
104	0	104	0	104	0	104	0	0		0	0	0
20	1	7	1	7	1	20	0	1	0	1	2	0
147	0	147	0	147	0	147	0	0		1	1	0
197	0	197	0	197	0	197	0	1	2	1	2	0
31	1	0	1			31	0	1	1	1	1	0
114	0	114	0	114	0	114	0	0		1	2	0
200	0	200	0	200	0	200	0	0		1	1	0
179	0	179	0	179	0	179	0	0		1	1	0
79	1	15	1	15	1	79	0	1	13	0	0	0
17	1	17	0	17	0	17	0	0		1	2	1

ACT yes or no	Type of ACT 4 groups	Days of ACT	Days from surgery til ACT	Reason for no ACT	Comorbiditi es, yes/no	Inflammato ry bowel disease	Cardiovasc ular	Lung disease (asthma, COPD)	Diabetes
0					1	0	1	0	0
0					1	0	1	0	1
0					1	0	1	0	0
0				1	1	0	1	0	0
0					0	0	0	0	0
0					1	0	1	0	1
0					1	0	1	0	1
0					1	0	1	0	0
0					1	0	1	0	0
0					0		0	0	0
0					0		0	0	0
0					1	0	1	1	1
0					1	0	1	0	1
0				1	1	0	1	0	0
0					0	0	0	0	0
0					1	0	1	0	0
0					0	0	0	0	0
0					1	0	1	0	0
0					1	0	1	0	0
0					0	0	0	0	0
0				1	0	0	0	0	0
0					1	0	1	0	0
0					1	0	1	0	1
0					1	0	1	0	0
0				1	1	0	0	0	1
0				1	1	0	1	0	0
0					1	0	1	0	0

0		1	0	1	1	0
0		0	0	0	0	0
0	1	1	0	1	0	0
0		1	0	1	0	0
0		0	0	0	0	0
0		1	0	1	0	1
0		0	0	0	0	0
0		0		0	0	0
0	8	0	0	0	0	0
0		0		0	0	0
0		0		0	0	0
0		1	0	1	0	0
0		1	0	1	0	0
0		1	0	1	0	0
0		1	0	1	0	0
0	1	1	0	1	0	1
0		1	0	1	0	0
0		0	0	0	0	0
0		1	0	1	0	0
0		0	0	0	0	0
0		1	0	1	0	0
0		1	0	1	1	1
0		1	0	1	0	0
0	3	1	0	1	0	0
0		0	0	0	0	0
0		1	0	1	0	0
0	1	1	0	1	0	0
0	0	1	0	1	0	0
0		1	0	1	0	0
0		1	0	1	0	0
0	3	1	0	1	0	0
0		1	0	1	0	0
0		1	0	1	0	0
0		0		0	0	0

0					1	0	1	0	0
0					0	0	0	0	0
0					1	0	1	0	0
0					0		0	0	0
0					0		0	0	0
0					0	0	0	0	0
0					1	0	1	0	0
0					0	0	0	0	0
1	1	155	54		0	0	0	0	0
0					1	0	1	0	0
0					1	0	1	0	1
0					1	0	1	0	0
0				1	0		0	0	0
0					1	0	1	0	0
0					0		0	0	0
0					0	0	0	0	0
0					0	0	0	0	0
0					0	0	0	0	0
0					1	0	1	0	0
0					1	0	1	0	0
0					1	0	1	0	0
0					0		0	0	0
0					0		0	0	0
0					1	0	1	0	0
0				0	0	0	0	0	0
1	1	166	44		0	0	0	0	0
0					1	0	1	0	0
1	1	148	47		0	0	0	0	0
0					0	0	0	0	0
0					1	0	0	0	1
0					1	0	1	0	0
0					1	0	1	0	1
0				0	0		0	0	0
0					0	0	0	0	0

0				1	1	0	1	0	0
0					1	0	1	0	0
1	2	106	70		1	0	1	0	0
0					0	0	0	0	0
0				0	1	0	1	0	1
0					0	0	0	0	0
0					0		0	0	0
0					0	0	0	0	0
0					0	0	0	0	0
0				1	1	0	1	0	1
1	1	126	55		0	0	0	0	0
0				1	0		0	0	0
0				1	0	2	0	0	0
1	1	126	56		0	0	0	0	0
0					0	0	0	0	0
0					1	0	1	0	1
0					0	0	0	0	0
0					1	0	1	0	0
0					1	0	1	0	1
0					0	0	0	0	0
1	1	125	57		1	0	1	0	0
1	1	176	48		0		0	0	0
0				1	0		0	0	0
0				1	0	0	0	0	0
0					0	0	0	0	0
0					1	0	1	0	1
0					0	0	0	0	0
0					1	0	1	0	0
0				7	1	0	1	0	0
0				1	1	0	1	0	0
0					0		0	0	0
0					0	0	0	0	0
0					1	0	1	0	0
0					1	0	1	0	0

0				1	0		0	0	0
0					1	0	1	0	0
0				1	1	0	1	0	1
0					1	0	1	0	0
0				6	0	0	0	0	0
0				1	1	0	1	0	0
0					1	0	1	0	0
0				2	0	0	0	0	0
1	2	155	46		1	0	1	0	0
1	1	120	47		1	0	1	0	0
1	2	14	42		1	0	1	0	0
0					0		0	0	0
1	1	105	51		0		0	0	0
0					0	0	0	0	0
0					1	0	0	0	1
0					1	0	1	0	0
0				1	1	0	1	0	0
0					1	0	1	0	0
1	1	57	68		0	0	0	0	0
0					1	0	1	0	0
0					0	0	0	0	0
0					1	0	1	0	1
1	1	120	39		1	0	1	0	0
0					1	0	1	0	0
0				4	0		0	0	0
0				1	1	0	1	0	0
0					0	0	0	0	0
1	2	134	33		0	0	0	0	0
0					0		0	0	0
0					1	0	1	1	0
1	1	140	43		1	0	1	0	0
1	1	159	63		1	0	0	1	0
1	1	163	49		1	0	1	0	0
0					0	0	0	0	0

1	1	161	63		1	0	1	0	0
1	1	130	49		1	0	1	1	0
0					1	0	1	0	0
0					1	0	1	0	0
0					0	0	0	0	0
0					0	0	0	0	0
1	2				1	0	1	0	0
1	2	160	56		0	0	0	0	0
1	1	127	48		0	0	0	0	0
1	1	90	89		1	0	1	0	1
1	1	127	49		0		0	0	0
1	1	152	51		1	0	1	0	0
0					0		0	0	0
1	1	161	59		1	0	1	0	1
0					0	0	0	0	0
1	1	138	50		1	0	1	0	0
0					0	0	0	0	0
0					1	0	1	0	1
1	1	12	59		0		0	0	0
0					1	0	0	1	0
1	1	171	115		0	0	0	0	0
0				7	0	0	0	0	0
0					0	0	0	0	0
1	2	91	281		1	0	1	0	0
1	2	161	50		0	0	0	0	0
0					0	0	0	0	0
1	2	66	49		1	1	1	0	0
0					1	0	1	0	0
1	1	137	76		0	0	0	0	0
0					1	0	1	0	1
0					1	0	1	0	0
0					0	0	0	0	0
0					1	0	1	0	0
0					0	0	0	0	0

1	1	99	57		1	0	1	0	1
0					1	0	1	0	0
0				3	1	0	1	0	0
0					0	0	0	0	0
0					1	0	1	0	0
0					1	0	1	0	0
0					1	0	1	0	0
0				1	1	0	1	0	0
0					1	0	1	0	1
0					0		0	0	0
0				1	1	0	1	0	0
1	2	53	53		0	0	0	0	0
0					1	0	1	0	0
1	1	36	55		1	0	1	0	0
0					1	0	1	0	0
1	2	131	51		0	0	0	0	0
0					0	0	0	0	0
1	2	63	64		1	0	1	1	0
0				5	0	0	0	0	0
0					1	0	1	0	0
0					0	0	0	0	0
1	1	162	117		1	0	1	0	1
1	1	77	191		0	0	0	0	0
0					1	0	1	0	0
1	1	154	59		0		0	0	0
0					0	0	0	0	0
0					1	0	1	0	0
0					0	0	0	0	0
1	1	167	47		1	0	1	1	0
1	2	147	55		1	0	1	0	1
1	2	105	58		0		0	0	0
0					0	0	0	0	0
1	2	178	54		0	0	0	0	0
1	1	167	57		1	0	1	0	1

1	1	142	51	1	0	1	0	0
1	1	169	43	0	0	0	0	0
1	2	56	58	0	0	0	0	0
1	1	105	44	0		0	0	0
0				0	0	0	0	0
0				1	0	1	0	0
1	0	168	52	1	0	1	0	0
1	1	151	58	0	0	0	0	0
1	1	192	59	0	0	0	0	0
0				0		0	0	0
0				0		0	0	0
1	1	169	45	1	0	1	0	0
0				0	0	0	0	0
0				0	0	0	0	0
1	2	160	92	1	0	1	0	0
0				1	0	1	0	0
0				0	0	0	0	0
0				1	0	1	0	0
1	2	133	57	0		0	0	0
1	2	14	58	1	0	1	1	0
0				0	0	0	0	0
1	2	7	69	1	0	1	0	0
1	2	183	53	0	0	0	0	0
1	1	147	72	0		0	0	0
0				1	0	1	0	0
1	2	34	49	1	0	1	0	0
0				0	0	0	0	0
0				0		0	0	0
1	2	123	358	1	0	1	0	0
0				0	0	0	0	0
0				1	0	0	1	0
0				1	0	1	0	1
1	1	162	40	0	0	0	0	0
0				0	0	0	0	0

0					0	0	0	0	0
0					1	0	1	0	0
0					0		0	0	0
0					0		0	0	0
0					1	0	1	0	0
1	1	183	40		0	0	0	0	0
0					0		0	0	0
0					0	0	0	0	0
1	2	144	48		1	0	1	0	1
1	1	147	57		0		0	0	0
0					0		0	0	0
1	1	99	63		0	0	0	0	0
1	1	139	34		1	0	1	0	0
1	1	140	55		1	0	1	0	0
1	2	148	49		0		0	0	0
1	3	42	179		0	0	0	0	0
1	2	104	76		1	0	0	0	1
0					1	0	1	1	0
0			6		0		0	0	0
1	2	186	44		0		0	0	0
1	2	91	56		1	0	1	0	1
0					1	0	1	0	0
0					0	0	0	0	0
0					0	0	0	0	0
0					1	0	1	0	1
1	2	171	74		1	0	1	0	0
0					0	0	0	0	0
0				1	1	0	1	1	1
1	1	151	43		0	0	0	0	0
0					0	0	0	0	0
1	1	149	54		0	0	0	0	0
0					0	1	0	0	0
0				1	1	0	1	0	0
0					0	0	0	0	0

0					0	0	0	0	0
0					0		0	0	0
0					0	0	0	0	0
1	4	100	68		0	0	0	0	0
0					0		0	0	0
0					0	0	0	0	0
0					0	0	0	0	0
1	2	119	58		0	0	0	0	0
1	2	103	42		0	0	0	0	0
0					1	0	1	0	0
0				6	0		0	0	0
0					0	0	0	0	0
0					0		0	0	0
0					0		0	0	0
0					0		0	0	0
1	2	188	48		0	0	0	0	0
0					1	0	1	0	0
1	2	161	58		1	0	1	0	0
0					0		0	0	0
1	1	52	72		0		0	0	0
0					0	0	0	0	0
1	2	149	48		0		0	0	0
0				7	0	0	0	0	0
0					0	0	0	0	0
0					1	0	0	0	1
1	2	176	58		0	0	0	0	0
1	1	196	344		0	0	0	0	0
1	2	91	52		0	0	0	0	0
1	1	45	218		0		0	0	0
1	2	157	59		0	0	0	0	0
1	2	148	53		0	0	0	0	0
1	2	168	78		1	0	1	0	0
1	2	119	63		1	0	0	1	0
1	2	53	42		0	0	0	0	0

1	2	111	50		0	0	0	0	0
1	2	105	45		0		0	0	0
1	1	179	71		0		0	0	0
0				1	0		0	0	0
0					1	0	1	0	0
0					0	0	0	0	0
1	2	84	84		0	0	0	0	0
0					1	1	1	0	0
1	2	147	32		0	0	0	0	0
1	2	155	57		0	0	0	0	0
0				1	1	0	1	0	1
1	2	110	66		0		0	0	0
0					0		0	0	0
0					0	0	0	0	0
1	2	168	80		0	0	0	0	0
0					0	0	0	0	0
0				7	0	0	0	0	0
0					0	0	0	0	0
0					0		0	0	0
0					0	0	0	0	0
1	2	109	68		0	1	0	0	0
0					1	2	1	0	1
0					0		0	0	0
0					1	0	0	0	1
0					0	0	0	0	0
1	2	172	29		0	0	0	0	0
1	4	112	44		0		0	0	0
0					0	0	0	0	0
1	1	161	53		0	0	0	0	0
1	2	176	51		0	1	0	0	0
0					0		0	0	0
1	3	1	68		0	2	0	0	0
