# Supplementary material for: The impact of adjuvant chemotherapy on survival in mucinous and non-mucinous rectal adenocarcinoma patients after TME surgery
Source: PLoS One. 2023 Feb 27;18(2):e0282211. doi: 10.1371/journal.pone.0282211 (PMC9970087; doi:10.1371/journal.pone.0282211)
Supplement: Attachment — Submitted filename: Rebuttal letter PLOS ONE 221215.docx [file pone.0282211.s002.docx]

Dr. Mona Pathak	Dec. 15^th^ 2022

Academic Editor

**PLOS ONE**

**Manuscript (MS) ID:** PONE-D-22-16390
**Manuscript title:** The impact of adjuvant chemotherapy on survival in mucinous and non-mucinous rectal adenocarcinoma patients after TME surgery

Dear Dr. Pathak,

Thank you very much for yours and the reviwers’ comments on our manuscript. We have carefully revised our MS according to the questions and suggestions. The detailed response to each suggestion is provided below. Two manuscripts are submitted, one with tracked changes and another without tracked changes.

Below, comments and suggestions are *italicized*, and our responses are marked in blue color for easier recognition.

**Comments and suggestions from Academic Editor PLOS ONE, Dr Mona Pathak**

*1. Please ensure that your manuscript meets PLOS ONE's style requirements, including those for file naming. The PLOS ONE style templates can be found at* [*https://journals.plos.org/plosone/s/file?id=wjVg/PLOSOne_formatting_sample_main_body.pdf*](https://journals.plos.org/plosone/s/file?id=wjVg/PLOSOne_formatting_sample_main_body.pdf) *and* [*https://journals.plos.org/plosone/s/fileid=ba62/PLOSOne_formatting_sample_title_authors_affiliations.pdf*](https://journals.plos.org/plosone/s/fileid=ba62/PLOSOne_formatting_sample_title_authors_affiliations.pdf)

Response: We would like to thank Editor Pathak for the valuable feed back on our manuscript.

We have carefully revised the manuscript according to the PLOS ONE´s style requirements with changes in the formatting due to previously overlooked errors.

We have also deleted the word “Disease-free survival” (page 5, line 102). Instead, the abbreviation DFS was used since disease-free survival was mentioned earlier (page 3, line 67).

*2. Thank you for stating the following in the Acknowledgments Section of your manuscript:*

*“This study was supported by grants from Region Östergötland and The foundation of the Department of Oncology in Linköping, Sweden.”*

*We note that you have provided funding information that is not currently declared in your Funding Statement. However, funding information should not appear in the Acknowledgments section or other areas of your manuscript. We will only publish funding information present in the Funding Statement section of the online submission form.*

*Please remove any funding-related text from the manuscript and let us know how you would like to update your Funding Statement. Currently, your Funding Statement reads as follows:*

*“NO -the authors recieved no specific funding for this work”*

*Please include your amended statements within your cover letter; we will change the online submission form on your behalf.*

Response: We have now removed the funding information from the Acknowledgement section and added the corrected funding-related text to the cover letter. We are grateful that you will change the online submission form on our behalf.

*3. We note that you have stated that you will provide repository information for your data at acceptance. Should your manuscript be accepted for publication, we will hold it until you provide the relevant accession numbers or DOIs necessary to access your data. If you wish to make changes to your Data Availability statement, please describe these changes in your cover letter and we will update your Data Availability statement to reflect the information you provide.*

Response: We have now added the relevant data in a PDF file.

*4. Please ensure that you include a title page within your main document. You should list all authors and all affiliations as per our author instructions and clearly indicate the corresponding author.*

Response: We have now added the title page to the main document. All authors and the corresponding affiliations have been listed as per PLOS ONE’s author instructions and the corresponding author is clearly indicated.

*5. Please review your reference list to ensure that it is complete and correct. If you have cited papers that have been retracted, please include the rationale for doing so in the manuscript text, or remove these references and replace them with relevant current references. Any changes to the reference list should be mentioned in the rebuttal letter that accompanies your revised manuscript. If you need to cite a retracted article, indicate the article’s retracted status in the References list and also include a citation and full reference for the retraction notice.*

Response: We have reviewed the Reference list carefully, there are no errors or retracted articles. The format has been changed to PLOS ONE formatting.

**Comments and suggestions from the Reviewer 1, dr Amine Souadka**

*First, I would like to thank the editor and the authors to the opportunity to review this well written article.*

*The authors from Sweden investigated a very interesting question, due to the lack of strength in the recommendation of adjuvant chemotherapy in stage 2 to 4 rectal cancer.*

*The authors succeed in exploring this research idea, using adequate statistical methods. However they need to correct thein conclusion and formulate them more cautiously.*

*Introduction*

*R1	The authors should focus more on the bad prognosis of MAC or undifferentiated rectal tumor adding even in complete response to neoadjuvant CRT, as shown in: 10.1186/s12885-019-6239-3 were in Dworack 4 patients, MAC and undifferentiated tumor were an independent factor of of unpaired disease free survival.*

Response: We would like to thank Dr. Souadka for the positive evaluation and valuable feed back to our manuscript. We have added the suggested reference to the Introduction section on page 3, lines 64-67.

*Study design*

*R1	The figure 1 should be in the results section not in the methods.*

**Response:** We have moved figure 1 and the corresponding text to the Result section on page 6, lines 118-123.

***Results***

*R1	The authors should report more details on the quality of surgery: total or partial mesorectal excision, completeness and radicality of the surgery , median circumferential margins, and information on surgeons and their expertise. All these informations may impact the survival of the patients independently of receiving or not adjuvante treatment.*

**Response:** All patients had surgery with **total** mesorectal excision (TME) as described in the section called

Surgery on page 9, line 158. The data on whether or not the surgery was radical is described in table 2, page

8. Unfortunately we do however not have the data on the median circumferential margin available even though

this would be interesting. Information regarding the surgeons expertise has been added on page 9, lines

158-159.

*R1	The rate of 10% of local recurrence after TME and preoperative RCC seems a little bit high. The authors should argue this result and give some explanations in the discussion part*

**Response:** Thirty-six patients (10%) had local recurrence after TME in our study. All patients but one had local recurrence before 84 months had passed since the diagnosis. At 24 months after surgery, 22 patients (6%) had had local recurrence and, by 48 months, 32 patients (9%) had had local recurrence. The result was comparable to available data, for example, the Dutch TME trial that had a total of 9% local recurrence including patients that both had and did not have radiotherapy (doi:10.1016/j.ejso.2009.11.011). We have added information about the timing of the recurrences on page 9, lines 161-163 and to the Discussion section, page 14, lines 246-249.

***Conclusion***

*R1	The consultation section should be written more cautiously in both the abstract and the manuscript:*

*The present study showed that RC patients with MAC may have better survival after adjuvant CT compared to those without adjuvant CT,*

*These results could possibly suppose that rectal MAC and NMAC patients may need different treatment strategies. and further comparative studies are needed..*

**Response:** We have changed the Conclusion section in the Abstract and in the manuscript on page 15, lines 314-317, according to the suggestions.

**Comments and Suggestions from the Reviewer 2, dr Atul Sharma**

*R2	Though this is a retrospective study but still gives and important message about role of adjuvant chemotherapy in MAC rectal cancer. Possibly because of small sample size of MAC in this retrospective analysis survival benefit between MAC and NMAC could not be show.*

*This gives rise to an important hypothesis for future trials where MAC may be treated with little more aggressive chemotherapy*

**Response:** We would like to thank Dr. Sharma for the valuable feed back on our manuscript.

Yours Sincerely,

Annica Knutsen (former last name Holmqvist), Associated Prof., MD. PhD.

Department of Oncology, and Department of Biomedical and Clinical Sciences

Linköping University

SE-581 83, Linköping

Sweden
